# Metallothionein expression on oysters ( *Crassostrea cuculata and Crassostrea glomerata*) from the southern coastal region of East Java

**DOI:** 10.12688/f1000research.17381.2

**Published:** 2020-04-30

**Authors:** Asus Maizar Suryanto Hertika, Kusriani Kusriani, Erlinda Indrayani, Defri Yona, Renanda Baghaz Dzulhamdhani Surya Putra

**Affiliations:** 1 Faculty of Fisheries and Marine Science, University of Brawijaya, Malang, East Java, 65165, Indonesia

**Keywords:** Heavy Metal, Biomarkers, Metallothionein, CLSM

## Abstract

**Background:** This study aimed to analyse levels of heavy metals (Pb, Hg and Cd) in the aquatic body, gills and stomach of the oysters *Crassostrea cuculata* and *Crassostrea glomerata*, the metallothionein (MT) level in the gills and stomach of both oysters, and relationships between heavy metals level (Pb, Hg and Cd) in the gills and stomach to MT level in both species of oysters.

**Methods:** The research method utilized was a descriptive method. The oyster samples were taken from three stations: Sendang Biru, Popoh and Prigi beaches. MT values were assessed using confocal laser scanning microscopy. The heavy metal levels were assessed using atomic absorption spectrophotometry method.

**Results:** Both oyster heavy metal content obtained in the southern coastal waters exceeded the safe limit set by the State Minister of Environment No. 51 of 2004. In general, the expression of MT was found to be higher in stomach tissue compared to gill tissue.

**Conclusions:** The levels of the heavy metals Pb, Hg, and has a strong relationship with MT levels in the gills and stomach in both types of oysters.

## Introduction

Coastal areas are often under the pressure of ecological pollution originating from human activities. One kind of pollutant is heavy metals, such as cadmium (Cd), Mercury (Hg), and lead (Pb), which originate from household and industrial waste effluent ^1, 2^. Heavy metals settle on the bottom of the seabed by sedimentation. This can contaminate marine biota with heavy metals and threaten human health as consumers ^3– 7^.

Metallothionein (MT) is a non-enzymatic protein in a low molecular weight which has a high cysteine content, does not have aromatic amino acids and is not heat-stable. The multiple thiol groups (-SH), formed by cysteine residues, allow MT to bind heavy metals ^8– 10^. MT has a specific metal binding ability. Each MT only binds one type of metal, with Cd M, Hg and Pb each binding a different MT ^11, 12^. MT has been widely used as a specific biomarker because the expression of MT reflects the presence of heavy metals ^13– 15^. Previous research has revealed that the induction of MT expression increases after the organism is exposed to heavy metals ^16^. Hertika *et al*. ^17^ found the existence of positive relationships between heavy metals and MT expression in North East coast oysters.

MT possess the ability to bind a certain amount metal in a cell and restore the ability to function of inactive proteins due to metal cadmium ^18^. According to Prabowo ^19^, heavy metals contained in waters can enter the body of aquatic biota. Heavy metals pass through the mouth and digestive organs, such as the surface of the gills. Therefore, organisms that live in waters with higher levels of heavy metal contamination will have higher heavy metal level.

Oysters, including benthic macrofauna species, are one of the best bioindicators of heavy metal contamination in an area ^20^. Oysters are potential biota contaminated by heavy metals, as these are filter feeders, and express MT, which is able to bind heavy metals Therefore, oysters can be used as test animals in monitoring the accumulation of heavy metal levels in polluted waters ^21^. This study aims to analyze heavy metal level (Pb, Hg, and Cd) in the tissues (gills and stomach) of oysters ( *Crassostrea cuculata* and *Crassostrea glomerata*) and the coastal waters of the South coast of East Java to determine their relationship to MT (MT) expression.

## Methods

### Sample collection

In total 108 oyster were used in this study. Three samples of oyster ( *Crassostrea cuculata* and *Crassostrea glomerata*) were collected in three of each of the three sub-stations on the Sendang Biru (Malang) coast, Prigi beach (Trenggalek) and Popoh beach (Tulungagung). Sub-station 1 located in port, sub-station 2 located in fish market, sub-station 3 located in between mangrove and beach. Sub-station 1, 2 and 3 on the Sendang Biru beach are geographically located at 8°26’01.3”S 112°41’01.8”E, 8°26’04.7”S 112°40’55.3”E and 8°25’48.2”S 112°41’17.0”E, respectively. Sub-stations 1, 2 and 3 of the Prigi coast are geographically located at 8°25’ 48.2”S 112°41’17.0”E, 8°15’47.9”S 111°48’11.6”E and 8°15’44.4”S 111°48’13.0”E, respectively. Sub-stations 1, 2 and 3 from Popoh beach are geographically located at 8°17’11.9”S 111°43’41.9”E, 8°17’13.2”S 111°43’47.2”E and 8°17’11.8”S 111°43’33.1”E, respectively. Oyster samples were taken three times for gills and stomach tissue taken in each sub-station and each was analyzed separately.

### Heavy metal analysis

Heavy metals (Pb, Cd, and Hg) in oysters (gills and stomach tissue) and the seawater at each sub-station were measured by atomic absorption spectrophotometry (AAS) following the measurement procedures in previous studies carried out by Hertika *et al*., 2018 ^17^ A total of 50 ml seawater samples obtained from each substation were filtered with a 0.45-mm polycarbonate membrane to separate particles which caused contamination in heavy metal measurements. Next, 1 M nitric acid was added to the water sample to obtain a pH value below 2.

The gill tissue and stomach taken from the oyster samples in each substation were prepared according to the method of Trinchella *et al*. ^22^. In order to obtain a complete oxidation process in the decomposition of organic substances, to each sample of gill and stomach tissue (0.2 grams), 2 ml of HNO _3_ were added. The samples were incubated for 30 minutes at low temperatures (5–8°C) to avoid minerals lost during the evaporation process. The sample is centrifuged for 15 minutes at 12,000 *g*. The supernatant produced from the centrifugation process was taken to measure the heavy metal content. Measurement of heavy metals (Pb, Cd, and Hg) was carried out using the A220 Atomic Absorption Spectrophotometer Variant (Variant, Inc.).

### Analysis of MT expressions

To analyse MT expression in this study, confocal laser scanning microscopy (CLSM) was performed (Confocal Olympus FluoView ^TM^ FV1000) based on previous research by Ockleford ^23^ and Mongan *et al*. ^24^. This observation system utilized a reverse-light-path fiber-optic signal that transmits Nomarski DIC signals to a second detector to visualize immunofluorescent and refractive index (RI) images. Images were observed using an Olympus U-TBI90 Microscope (Olympus, Japan) and inputted to Olympus Fluoview v4.2a, Japan for calculating MT expression quantities. Briefly, the gills and stomach in oyster samples ( *Crassostrea cuculata* and *Crassostrea glomerata*) were preserved into 10% formaldehyde. The sample was cut into 2–3 mm sections using a microtome and dehydrated using the Tissue Tex Processor. The samples were twice soaked with xylol (#CAT 1086612511, Merck, Japan) for 10 minutes each. Then the sample was fixed with absolute ethanol 90%, for 5 minutes. Immediately, the sample was soaked with 10 mM pH 6 buffer citrate for 15 minutes. Samples were blocked with PBST containing BSA 2% (CAT# 15561020, Thermo Fisher, USA) for 1 hour in room temperature. Furthermore, the sample was labelled with the Anti-MT Primary monoclonal mouse Antibody (1:1000, CAT# UC1MT, Gene Tex, USA) which contained 2% BSA for 1 hour at room temperature. Samples were rinsed with PBST for 8 minutes. Furthermore, the sample is labeled with rhodamine-conjugated Mouse IgG Antibody (CAT#610-1002, ROCKLAND Immunochemical Inc,USA) containing 2% BSA for 1 hour at room temperature. The sample was rinsed using PBST and dried. Lastly, glycerol was added into the sample and observed using CLSM.

### Water quality measurement

Water quality in this study was measured based on standard methods ^25^. Dissolved oxygen concentration was measured *in situ* using an oximeter (YSI PRO 20). The pH was also measured *in situ* using a pH pen (PH 2011 ATC) at each sub-station. Temperature and salinity were measured using a mercury thermometer and refractometer (RHS-10ATC, SINOTECH), respectively.

### Data analysis

This study used regression correlation analysis with a simple linear regression model in SPSS version 16.0 software. Professional charts are created using the GraphPad Prism 7.00 application. Using the method outlined bn Hertika *et al.* ^17^, the relationship between heavy metal levels with MT (MT) expression was obtained from multiple regression results with variable Y exhibiting heavy metals in oyster gills or stomach tissue. Variable X exhibited levels of Pb, Cd, and Hg.

## Results and discussion

### Water quality analysis

**Figure 1.**
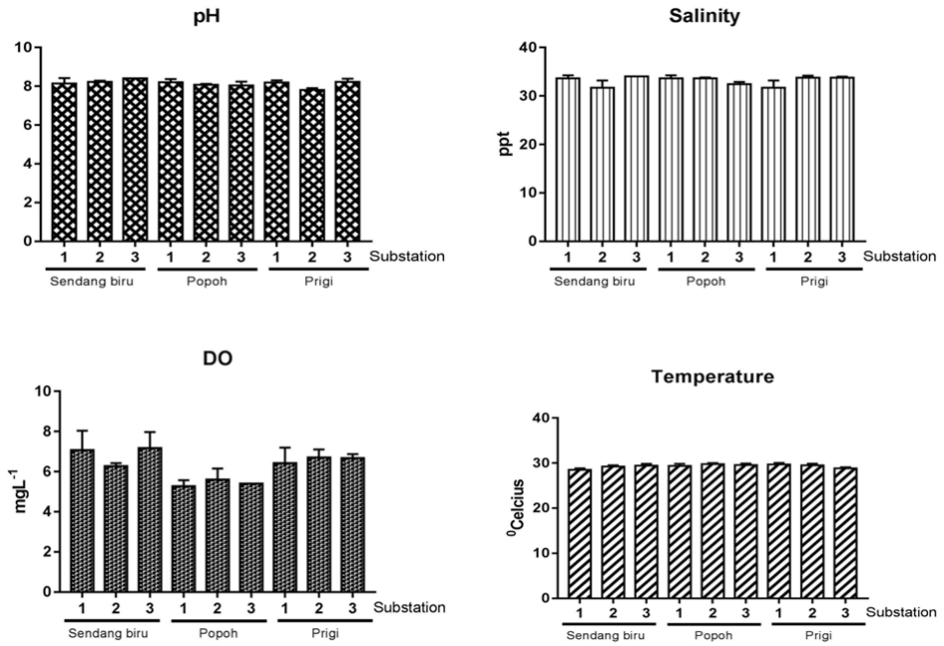
Analysis of water quality at the three research stations.

The research station was taken from three different locations. The first station is Sendang Biru Coastal area, one of the major ports on the southern coast of East Java and tourist area. The second station is the Popoh Beach area which is a tourist area and settlement. The third station is Prigi Beach which is a tourist beach, settlement and fishing port area. We observed physical and chemical water quality parameters that support the life of *Crassostrea cuculata* and *Crassostrea glomerata*, namely temperature, acidity (pH), dissolved oxygen (DO) and salinity ( Figure 1).

Water quality monitoring exhibited that there is no significant difference between each station and exhibited that the water quality is good for the oyster ecology. According to KEPMENLH. 51 of 2004 ^26^, it indicates the temperature suitable for oysters growth is 25–34°C. Furthermore, the pH level suitable for oysters’ ecosystem ranging from 6.8 to 8.8. DO levels of more than 5 mg l ^-1^ are required to support aquatic organisms’ survival ^26^.

### Heavy metal levels in the waters

**Figure 2.**
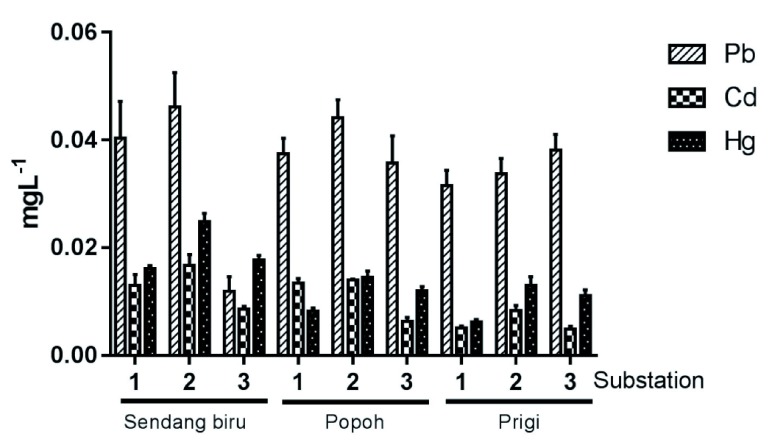
Analysis of Pb, Cd and Hg levels in seawater samples from each substation of Sendang Biru, Popoh, and Prigi stations.

Analysis of the Pb, Cd and Hg content at the three research stations (Sendang Biru, Popoh, Prigi) is shown in Figure 2. The range of highest Pb concentrations in each substation was 0.03–0.054 mg l ^-1^; the highest Hg concentration was 0.01–0.026 mg l ^-1^ and that of Cd was 0.009–0.018 mg l ^-1^. The highest concentration of Pb, Cd and Hg heavy metals were found at sub-station 2 in Sendang Biru for 0.054, 0.018, 0.026 mg l ^-1^. In general, some measured heavy metal content has passed the specified quality standard. Based on the Decree of the State Minister of Environment No. 51 of 2004 concerning seawater quality standards for heavy metal content ^26^, the Hg content appropriates the aquatic environment must not exceed 0.003 mg l ^-1^, Pb 0.05 mg l ^-1^ and Cd 0.01 mg l ^-1^.

### Heavy metal in the gills and stomach tissue

**Figure 3.**
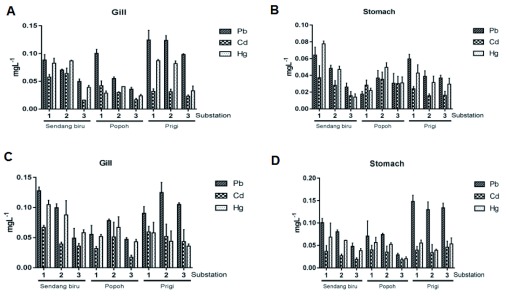
Pb, Cd, and Hg levels in the gills and stomach of ( **A**, **B**) *Crassostrea cuculata* and ( **C**, **D**) *Crassostrea glomerata* at Sendang Biru, Popoh and Prigi stations.

Heavy metals (Pb, Cd, and Hg) in *Crassostrea cuculata* and *Crassostrea glomerata* gills and stomach tissue is exhibited in Figure 3. The highest Pb content in the gills of *Crassostrea cuculata* was obtained at the Prigi station, at sub-station 1 at 0.13 mg l ^-1^, the highest Cd and Hg concentrations were obtained from Sendang Biru station in sub-station 2 at 0.08 mg l ^-1^ and 0.09 mg l ^-1^, respectively ( Figure 3A). Whereas the highest heavy metal level in *Crassostrea cuculata* stomach tissue Pb, Hg and Cd levels were observed at the Sendang Biru station in sub-station 1, at 0.067, 0.036 and 0.077 mg l ^-1^ respectively ( Figure 3B).

Furthermore, the heavy metal content in the gills of *Crassostrea glomerata* is exhibited in Figure 3C. The highest Pb, Hg, and Cd values were obtained at Sendang Biru station at sub-station 1, at 0.142, 0.071 and 0.11 mg l ^-1^, respectively. The highest value of stomach Pb and Cd content was observed in Prigi stations in substations 1 and 3: 0.145 and 0.047 mg l ^-1^, respectively ( Figure 3D). The highest value of Hg was obtained at Sendang Biru station at sub-station 1, which was 0.078 mg l ^-1^.

The accumulation of heavy metals in this study exhibit the same pattern in the study conducted by Bilgin *et al*. ^27^, which found that accumulation of heavy metals in the soft tissue of mollusks, Pb was found higher than Cd or Hg. Aquatic organisms are capable of absorbing and accumulating heavy metals in several ways: through the respiratory tract (gills), digestive tract, and skin surface diffusion ^28^. In this study, the highest accumulation of heavy metals was found in gill tissue. According to Hutagulung ^29^, the high accumulation of heavy metals in gills is closely related to the nature of biota. The oyster’s food intake is conducted through filtering water (filter feeders). Furthermore, Soto *et al*. ^30^ revealed that gills are the main target tissue for absorption contamination of dissolved heavy metal ions in aquatic bodies.

Research result exhibited that *Crassostrea cuculata* and *Crassostrea glomerata* have different values of heavy metals as each organism has a different ability to accumulate heavy metals. Based on the results of the study by Fattorini *et al*. ^31^ *Mytilus galloprovincialis* is able to accumulate Pb, Cd and Hg heavy metals at 0.29–2.95 mg l ^-1^, 0.41–1.60 mg l ^-1^, and 0.02–0.19 mg l ^-1^, respectively. However, Kucuksezgin *et al*. ^32^ achieved different results; *Thylacodes decussatus* was observed to absorb Pb at levels ranging from 0.38–1.2 mg l ^-1^, Cd at 0.03–0.24 mg l ^-1^, and Hg at 0.04–0.13 mg l ^-1^. This may be related to the tendency of specific bioaccumulation of bivalves, based on different habitats, lifestyles, and abundance of food. Some studies emphasize that metal accumulation has presented different species-specific capacities for bivalves ^33, 34^. It is claimed that this difference is related to the metabolic rate of bivalve species ^35^. The bioaccumulation pattern of metals can generally be attributed to the presence of anthropogenic inputs or lithogenic sources affecting the area. Seasonal variations in bivalves metal concentrations result from many factors, such as large differences in water temperature, particulate metal runoff to coastal waters, food availability. It is caused by transferring metals from water to feeding-filtering organisms, body weight changes during gonadal development, and biomass release associated with sexual reproduction ^36– 38^. For bivalves, the accumulated changes depend on the metal and the ability of different species or genera to store or/and remove metals from the tissue. In general, metal concentrations in bivalves increase with increasing shell size; however, in some cases, metal concentrations may decrease due to the detoxification process in these organisms ^39, 40^. Raw data are available on OSF ^41^.

### MT levels in the gills and stomach of *Crassostrea cuculata*

**Figure 4.**
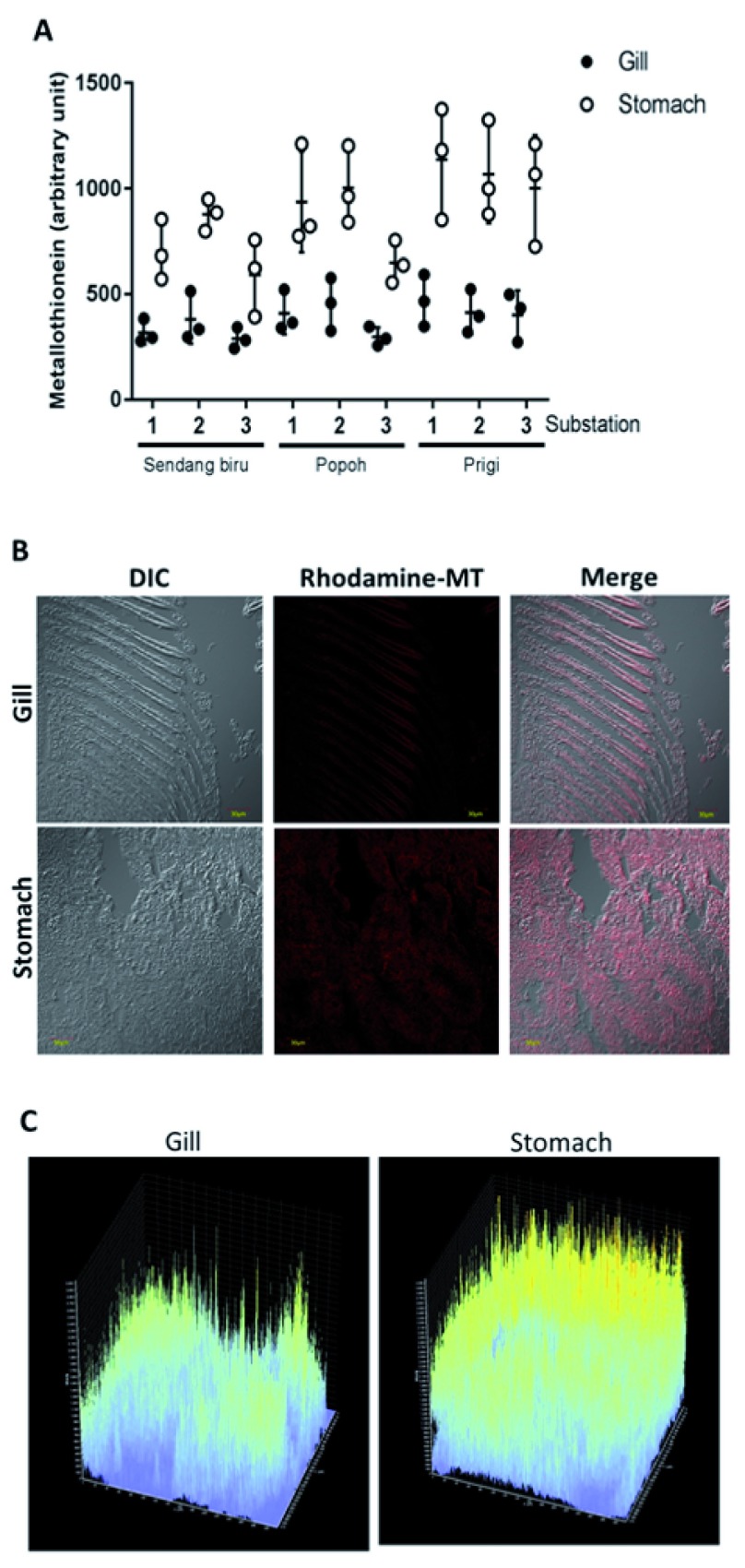
Metallothionein (MT) expression in *Crassostrea cuculata* gills and stomach. ( **A**) The quantity of MT expression at each station. ( **B**) Morphology of MT expression in gills and stomach, ( **C**) Absorbance gating of MT expression from a representative experiment.

MT content analysis is exhibited in Figure 4. Research results exhibited that in MT stomach tissue overall expression was higher than gill tissue ( Figure 4A). The highest metallothionine expression in gill tissue was obtained at Prigi station at 810.876–1387.61 arbitrary units. The lowest expression on the stomach tissue was found at Sendang Biru station ranging from 453.246–511.098 arbitrary units. Highest MT expression in gill tissue was also found at Prigi station ranging from 325.976–622.534 arbitrary units. On the other hand, the lowest value was obtained at the Sendang Biru station at 276.254–498.512 arbitrary units. MT expression in the stomach tissue is higher than that in the gill tissue. This is supported by assessment of the morphology of MT expression using rhodamine-labelled MT in the gill and stomach tissue. In Figure 4B, Rhodamine-MT as metallothionine marker is expressed brighter in stomach tissue compared to gill tissue. Figure 4C shows rhodamine-MT absorption, as an MT marker, is recorded to have a higher intensity in stomach tissues compared to gill tissues.

Heavy metal content was inversely proportional to MT expression in the gill and stomach tissues. The high content of heavy metals in the gills stimulates high MT expression to bind and detoxify heavy metals quickly. Therefore, MT quantity detected on the tissue decreases. Conversely, heavy metal in stomach tissue accumulates less compared to gill tissue. Hence, the detoxification process is slower. It indicates that MT quantity detected in this study is higher. According to Ringwood *et al*. ^42^, there is a positive relationship between MT and heavy metal pollutants. Heavy metal contaminants can cause systemic damage to an organism and result in increased MT production. Previous research has revealed that MT has a crucial role in various processes of biological activity; it binds heavy metals and conduct recovery process from systemic damage caused by heavy metals through homeostasis process (dynamic balancing of the body’s biological processes) to heavy metals ^43, 44^, and heavy metal detoxification ^45, 46^. The function of MT in heavy metal detoxification mainly depends on the high-affinity bond between heavy metals and MTs, which causes heavy metal absorption to be higher than that of important macromolecules ^45, 47^. It indicates that MT plays an important role in protecting cells from heavy metal poisoning ^48– 51^. It is proven that MT could be a biomarker useful for predicting heavy metal toxicity and heavy metal detoxification toxic to organisms ^52, 53^. Raw data are available on OSF ^41^.

### Analysis of MT levels in *Crassostrea glomerata* gills and stomach

**Figure 5.**
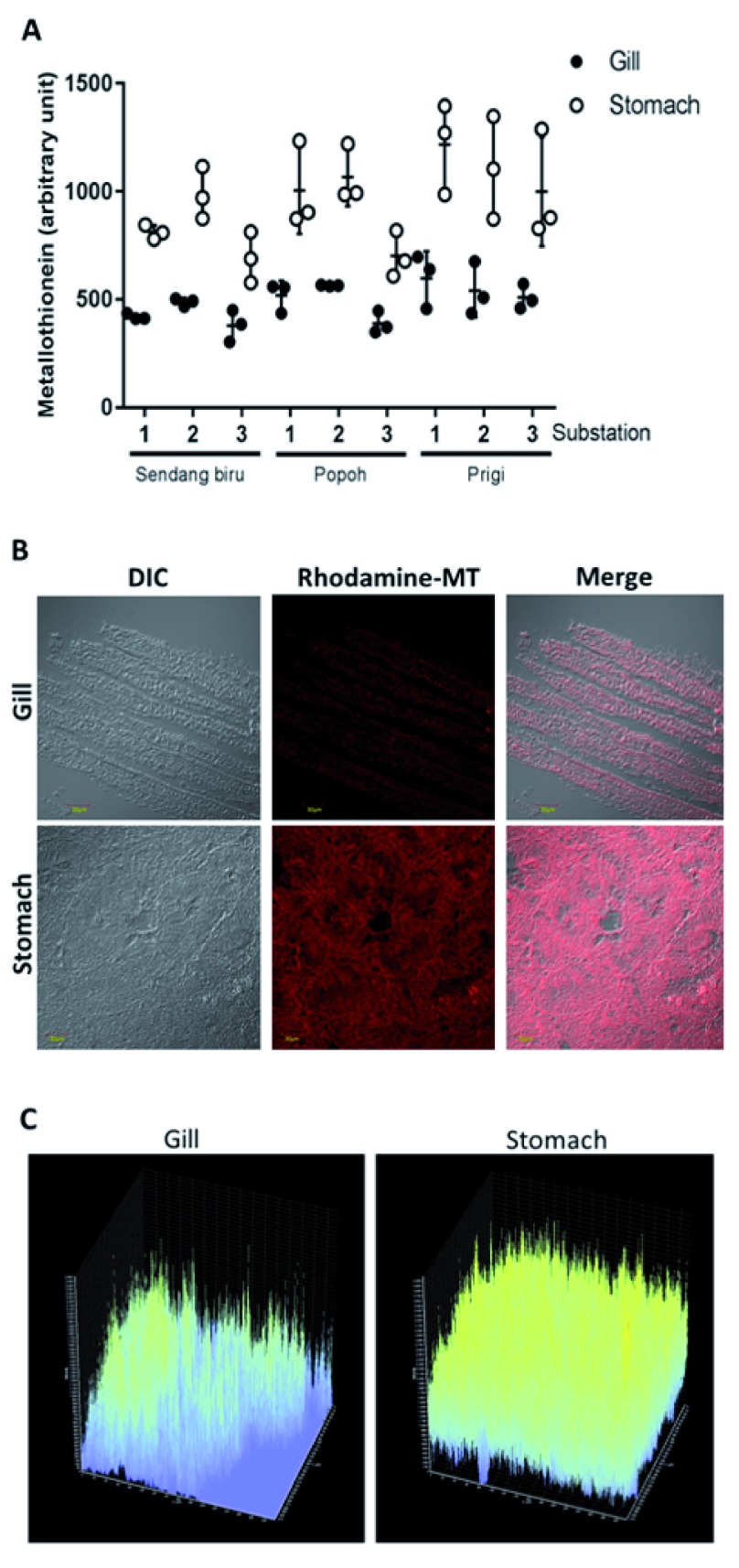
Metallothionein (MT) expression in gills and stomach of *Crassostrea cuculata*. ( **A**) The quantity of MT expression at each station. ( **B**) Morphology of MT expression in gills and stomach. ( **C**) Absorbance gating of MT expression from a representative experiment.

MT content analysis is exhibited in Figure 5. Similar results were obtained in the analysis of *Crassostrea glomerata* gills and stomach. MT expression in the stomach tissue of *Crassostrea glomerata* is expressed higher than gill tissue ( Figure 5A). The highest MT expression in stomach tissue was obtained at Prigi Station sub-station 1 with a value of 1412,112 arbitrary units. The lowest MT expression in stomach tissue was obtained from Sendang Biru sub-station 3 with a value of 576,243 arbitrary units. Furthermore, the highest MT expression in gill tissue was obtained from Prigi substation 1 with a value of 756,381 arbitrary units. The lowest MT tissue gill expression was obtained at Sendang Biru substation 3, with a value of 366,125 arbitrary units. Higher MT expression was observed in stomach tissue compared to gill tissue morphologically ( Figure 5B). The morphological results exhibited that MT labeled Rhodamine-B in stomach tissue appears brighter than gill tissue. Rhodamine-MT is a MT marker used in this study. Figure 5C exhibited that the Rhodamine-MT absorption as an MT marker possesses a higher intensity in gill tissue compared to stomach tissue.

Rumahlatu, *et al*. ^18^ stated that MT protein which acts as a metal binding protein can be used as an indicator of pollution, as the presence of MT in oysters serves as a binder of heavy metals that accumulate in the body. Based on the research result, MT expression in *Crassostrea glomerata* and *Crassostrea cuculata* has different results. According to Tapiero and Tew ^54^, MT expression levels vary between species, these levels are determined by the identity of metal atoms bound to proteins, and the difference in metal distribution between MT isoforms. This may affect MT expression levels, therefore indicating that MT is involved in cellular homeostatic control and element regulation. MT expression in *Crassostrea cuculata* highest value was found in stomach tissue and the lowest value in gill tissue. This is inversely proportional to the heavy metal content, which wsa highest in the gills. In this case, the high content of heavy metals in the gills causes high MT production, which in turn is rapidly used for homeostasis and detoxification from damage caused by these heavy metal toxins. Therefore, MT expression in the gill tissue was detected as lower than that in stomach tissue. In previous studies, MT participated in metal ion homeostasis and detoxification, and anti-oxidative damage ^55– 57^. Furthermore, MT expression is governed by the rate of accumulation of heavy metals, and MT plays an important role in metal detoxification and homeostasis ^58, 59^. Some species develop physiological adaptations to tolerate metal pollutants ^60^ which use two major detoxification mechanisms. The oyster uses metal binding compounds in the cytosol, such as MT (or similar proteins), or mineralization of minerals ^61^. The relativity of these two detoxification mechanisms varies greatly depending on the species and habitat. According to Amiard *et al*. ^61^, a decrease in MT concentration in organisms accumulated by heavy metals is influenced by cytotoxic effects in the detoxification process. Should an organism accumulate high heavy metals, a significant reduction in MT is caused as it is used in the process of suppressing the reactive production of ROS species oxygen responsible for oxygen metabolism ^62^.

### The relationship between Pb, Cd, and Hg levels and MT expression in *Crassostrea cuculata* gills and stomach

The relationship between heavy metal level Pb, Hg, and Cd with MT levels in *Crassostrea cuculata* gills exhibited a very strong value with the coefficient of determination (R ^2^) of 0.908. Based on the results of multiple linear regression equations of heavy metal level in the aquatic body against MT levels in *Crassostrea cuculata* gill tissue, the following formula was used Y = 242.337 + 2,128.234 X _1_ + 88.354 X _2_ + 2,182.218 X _3_. These results indicate that a 1 ppm Pb ( *X _1_*) increase will increase MT expression 2,128,234 arbitrary units. Should Cd increased by 1 ppm ( *X _2_*), it will increase MT expression by 88,354 arbitrary units. On the other hand, a 1 ppm Hg ( *X _3_*) increase would increase the MT expression of 2,182,218 arbitrary units.

Furthermore, a similar result was found in the relationship between the heavy metal level of Pb, Hg, and Cd with the stomach tissue MT expression. It indicates a strong relationship with the value of the coefficient of determination (R ^2^) of 0.92. Multiple linear regression equations of heavy metal level in the aquatic body against MT levels *Crassostrea cuculata* stomach assessment obtained the equation Y = 494.528 + 4,075.811 X _1_ + 2,852.821 X _2_+ 5,990.359 X _3_. The equation exhibited that Pb (X1) 1 ppm increase would, in turn, increase MT expression 4,075,811 at arbitrary units. Cd (X2) 1 ppm increase would increase MT expression 2,852,821 at arbitrary units. Hg (X3) when rising 1 ppm increase will increase MT expression of 5,990,359 arbitrary units.

Hasan *et al*. ^63^ stated that when the accumulation of heavy metals in the body of shellfish increases the synthesis of MT will probably reach the maximum level. The research conducted by Li *et al*. ^64^, exhibited a positive correlation between Cd heavy metal and MT levels in the gills and mantle of the bivalve group, which means that MT can be used as a biomarker for Cd heavy metal pollution. Furthermore, Sakulsak *et al*. ^65^ stated that the occurrence of exposure to heavy metals and the accumulation of heavy metals in cells can increase MT levels in tissues. Hence, MT can be used as a biomarker in environmental toxicology.

### The relationship between Pb, Cd, and Hg levels and MT expression in *Crassostrea glomerata* gills and stomach

A very strong relationship was obtained in Pb, Hg, Cd heavy metal level and gill MT expression of *Crassostrea glomerata* with a coefficient of determination (R2) of 0.943. Multiple linear regression equations of heavy metal level in aquatic body against MT expression of *Crassostrea glomerata* gills is Y = 320.254 + 2,311.778 X _1_ + 910.719 X _2_ + 2,173.765 X _3_. This equation exhibited that a 1 ppm increase in Pb (X _1_) will increase MT expression 2,311,778 arbitrary units. Furthermore, a 1 ppm increase in Cd (X _2_) will increase MT expression 910,719 arbitrary units. A 1 ppm increase in Hg (X _3_) will cause an increase in MT expression at 2,173,765 arbitrary units.

The relationship between the heavy metal level of Pb, Hg, Cd in the aquatic body and MT expression *Crassostrea glomerata* stomach tissue exhibited a strong relationship with the value of the coefficient of determination (R ^2^) of 0.918. Multiple linear regression equations of heavy metal level in the aquatic body against MT expression in *Crassostrea glomerata* stomach tissue was found to be similar. Y = 570.492 + 4,603.743 X _1_ + 3,455.676 X _2_+ 4,333.870 X _3_. The equation exhibited Pb (X _1_) up 1 ppm increase will cause an increase in MT expression of 4,603,743 arbitrary units. Furthermore, a 1 ppm increase in Cd (X _2_) will cause an increase in MT expression of 3,455,676 arbitrary units. A 1 ppm increase in Hg (X _3_) will result in an increase in MT expression of 4,333,870 arbitrary units.

According to Rumahlatu, *et al*., ^18^, MT acts as a metal-binding protein. It can be used as an indicator of pollution, as the presence of MT in oysters serves as a binder of heavy metals that accumulate in the body. Although many species can produce MT, oysters have exhibited a higher accumulation rate for metals compared to other species because they are filter feeders and tend to settle in one place ^66^. MT can bind metals very strongly, but exchanging bonds with other proteins may take place easily. MT bonds to metals possess high thermodynamic stability but low kinetic stability ^67^.

## Conclusion

Based on the results of the study it can be concluded that the heavy metal levels in the three locations assessed (Sendang Biru, Popoh, and Prigi) have exceeded the specified quality threshold. Furthermore, the relationship between Pb, Hg, and Cd heavy metal level in the aquatic body has a strong relationship with the expression of MT in oysters’ stomach and gills ( *Crassostrea cuculata* and *Crassostrea glomerata)*.

## Data availability

Raw data from the present study, including heavy metal levels in all oyster samples and all raw immunofluorescent images, are available on OSF. DOI: https://doi.org/10.17605/OSF.IO/37BVQ ^41^.

Data are available under the terms of the Creative Commons Zero "No rights reserved" data waiver (CC0 1.0 Public domain dedication).
