# Supplementary material for: Metallothionein expression on oysters ( Crassostrea cuculata and Crassostrea glomerata) from the southern coastal region of East Java
Source: F1000Res. 2020 Apr 30;8:56. Originally published 2019 Jan 14. [Version 2] doi: 10.12688/f1000research.17381.2 (PMC7217233; doi:10.12688/f1000research.17381.2)
Supplement: Peer review history

# Peer review: Reviewer response for version 2

**DOI:** 10.5256/f1000research.26095.r62936

**Published:** 2020-05-11

**Authors:** Rossita Shapawi

**Affiliations:** 1 Borneo Marine Research Institute, Universiti Malaysia Sabah, Kota Kinabalu, Malaysia

Is the work clearly and accurately presented and does it cite the current literature?

Yes

If applicable, is the statistical analysis and its interpretation appropriate?

Yes

Are all the source data underlying the results available to ensure full reproducibility?

Yes

Is the study design appropriate and is the work technically sound?

Yes

Are the conclusions drawn adequately supported by the results?

Yes

Are sufficient details of methods and analysis provided to allow replication by others?

Yes

Reviewer Expertise:

NA

I confirm that I have read this submission and believe that I have an appropriate level of expertise to confirm that it is of an acceptable scientific standard.

# Peer review: Reviewer response for version 1

**DOI:** 10.5256/f1000research.19007.r57619

**Published:** 2020-01-29

**Authors:** Rossita Shapawi

**Affiliations:** 1 Borneo Marine Research Institute, Universiti Malaysia Sabah, Kota Kinabalu, Malaysia

**Title **

- I would suggest a title that contain "heavy metal" term in it. Not everyone is familiar with the word metallothionein. e.g.  "Determination of heavy metal contents of oysters ( *Crassostrea cuculata* and *Crassostrea glomerata*) from the .....using metallothionein expression method"

**Abstract**

- Background - ...MT level in both types of oyster (replace types with species).
- Conclusions - The sentence needs to be improved - Eg. The level of heavy metals ...has a strong relationship with MT...

**Introduction**

- This section requires language editing.  You also need to mention the purpose of comparing two different species of oysters in this study. 

**Methods**

- How many samples (n) altogether were used in the study?  I cant find any information on this. The first line stated that three samples of oysters were used? Were these three groups of samples?
- Several typo/formatting issues here to be fixed.  

**Results and Discussion**

- Similarly, this section also requires language editing.  
- You mentioned that your findings in  soft tissue exhibit the same pattern with Bilgin et al.  - Pb>Cd>Hg???  Figure 2 shows Pb>Hg>Cd ????
- There is no discussion on the background of the sampling areas.  Any particular differences among the three areas (Eg. industrial activities) that may influence the results?
- I suggest the "Water quality analysis"  to be presented before other results and discussion.  

**General**

- The findings of the present study is considered very significant to the local community. I recommend this manuscript to be accepted for publication with minor corrections.

Is the work clearly and accurately presented and does it cite the current literature?

Yes

If applicable, is the statistical analysis and its interpretation appropriate?

Yes

Are all the source data underlying the results available to ensure full reproducibility?

Yes

Is the study design appropriate and is the work technically sound?

Yes

Are the conclusions drawn adequately supported by the results?

Yes

Are sufficient details of methods and analysis provided to allow replication by others?

Yes

Reviewer Expertise:

Aquaculture, Aquaculture feed development, fish nutrition, applied phycology

I confirm that I have read this submission and believe that I have an appropriate level of expertise to confirm that it is of an acceptable scientific standard, however I have significant reservations, as outlined above.

# Response

**Published:** 2020-04-24

**Authors:** Asus Hertika

**Affiliations:** Lecturer at brawijaya university, Indonesia

**1. Inquiry**

I would suggest a title that contain "heavy metal" term in it. Not everyone is familiar with the word metallothionein. e.g.  "Determination of heavy metal contents of oysters (Crassostrea cuculata and Crassostrea glomerata) from the .....using metallothionein expression method"

**Response:** Thanks for the reviewer's suggestion. We prefer to use this title to reviewer title suggestion. The word “determination of heavy metal….using metallothionein methods” in reviewer suggestion is not proper. Because we can’t determine heavy metal content using metallothionein. However, our title more proper to describe the article.. 

**2. Inquiry**

Background - ...MT level in both types of oyster (replace types with species).

Conclusions - The sentence needs to be improved - Eg. The level of heavy metals ...has strong relationship with MT...

**Response: **Thank you very much for your concern and suggestion. In the background of abstract, we already replace types with species. Furthermore, in the conclusion of abstract we have changed the sentence. 

**3. Inquiry**

This section requires language editing.  You also need to mention the purpose of comparing two different species of oysters in this study. 

**Response: **The reviewer raises an interesting concern. The purpose of comparing two different species of oysters in this study detail already put.

**4. Inquiry**

How many samples (n) altogether were used in the study?  I cant find any information on this. The first line stated that three samples of oysters were used? Were these three groups of samples?

Several typo/formatting issues here to be fixed.  

**Response:** Thank you very much for your valuable suggestion. We have written the detailed total sample we have fixed several issues or typo. There are three groups sample from different location that the oyster was taken. They are sendang biru (group 1),  Prigi Beach (Group 2), Popo beach (group 3). In each group have 3 substation sampling. In this study there are 108 oyster in total was used.

**5. Inquiry**

Results and Discussion

Similarly, this section also requires language editing.  

You mentioned that your findings in  soft tissue exhibit the same pattern with Bilgin et al.  - Pb>Cd>Hg???  Figure 2 shows Pb>Hg>Cd ????

**Response:** the reviewer raises an interesting concern and valuable suggestion. Thanks for the question. In this case, the result showed that the content of Pb was higher than Cd and Hg, however, It still has same result with bilgin et al that Pb has higher content than Cd and Hg. Further, we have made the new sentence of this already. 

**6. Inquiry**There is no discussion on the background of the sampling areas.  Any particular differences among the three areas (Eg. industrial activities) that may influence the results?

**Response: **The reviewer raises an interesting concern and valuable suggestion. Each sampling area has difference characters. We have put the detailed the differences among the three areas. 

**7. Inquiry**

I suggest the "Water quality analysis"  to be presented before other results and discussion.

**Response:** The reviewer raises an interesting concern. We have put  the water quality analysis at the before other results and discussion follow reviewer suggestion.

# Peer review: Reviewer response for version 1

**DOI:** 10.5256/f1000research.19007.r43030

**Published:** 2019-02-04

**Authors:** Ima Yudha Perwira

**Affiliations:** 1 Aquatic Resource Management, Faculty of Marine Science and Fisheries, Udayana University, Jimbaran, Indonesia

**Review:**

 This paper explains about the usage of Metallothionein (MT) expression as a specific biomarker for the presence of cadmium (Cd), mercury (Hg), and lead (Pb) in the tissues (gills and stomach) of oysters ( *Crassostrea cuculata* and *Crassostrea glomerata*) and the coastal waters of the South coast of East Java. This study has used the appropriate methodologies to measure several parameters. The heavy metals (Pb, Cd, and Hg) are measured using atomic absorption spectrophotometry (AAS). Heavy metals in the water samples are directly measured by using AAS, while that in the gill tissue and stomach are extracted (using nitric acid and incubated at 5-8 ^o^C for 30 minutes) before the measurement. The expression of MT is observed by using confocal laser scanning microscopy (CLSM). The authors also mentioned the detailed measurement of water quality for supporting the data of this study. The result of this study showed that the heavy metal content in the water is generally past the specific quality standard (based on the Decree of the State Minister of Environment No. 51 of 2004 concerning seawater quality standards for heavy metal content) (Jelaskan kenapa bisa tinggi). The analysis of MT expression on both gill tissue and stomach showed the highest value in the Prigi station. This study also showed a strong relationship between heavy metals (Pb, Cd, and Hg) with gill tissue and stomach in both *Crassostrea cuculata* and *Crassostrea glomerata.*

**Comments:**

 This study is interesting, but some points need to be considered. It would be better for the authors to mention some previous study about the using of MT expression as biomarker in the marine species. This study is also interesting because it was carried out in the south coastal area of Java Sea. There is industrial activity located in this area. It would be nice if the authors could mention the importance of doing this study in that area. The highest heavy metal content in the gill tissue and stomach of oysters is observed in the Prigi and Sendang Biru stations. The author has compared the heavy metal content in the oyster with Mytilus and Thylacodes. It would be better if there is another comparative result on the oyster.

Is the work clearly and accurately presented and does it cite the current literature?

Yes

If applicable, is the statistical analysis and its interpretation appropriate?

Yes

Are all the source data underlying the results available to ensure full reproducibility?

Yes

Is the study design appropriate and is the work technically sound?

Yes

Are the conclusions drawn adequately supported by the results?

Yes

Are sufficient details of methods and analysis provided to allow replication by others?

Yes

Reviewer Expertise:

Water polution and ecotoxicology

I confirm that I have read this submission and believe that I have an appropriate level of expertise to confirm that it is of an acceptable scientific standard.
